# Influence of respiratory motion management technique on radiation pneumonitis risk with robotic stereotactic body radiation therapy

**DOI:** 10.1002/acm2.12338

**Published:** 2018-04-26

**Authors:** Christopher H. Chapman, Christopher McGuinness, Alexander R. Gottschalk, Sue S. Yom, Adam A. Garsa, Mekhail Anwar, Steve E. Braunstein, Atchar Sudhyadhom, Paul Keall, Martina Descovich

**Affiliations:** ^1^ Department of Radiation Oncology University of California San Francisco CA USA; ^2^ Sydney Medical School University of Sydney Camperdown Australia

**Keywords:** computer‐assisted, dose–response relationship, image‐guided, radiation, radiation pneumonitis, radiosurgery, radiotherapy, radiotherapy planning

## Abstract

**Purpose/Objectives:**

For lung stereotactic body radiation therapy (SBRT), real‐time tumor tracking (RTT) allows for less radiation to normal lung compared to the internal target volume (ITV) method of respiratory motion management. To quantify the advantage of RTT, we examined the difference in radiation pneumonitis risk between these two techniques using a normal tissue complication probability (NTCP) model.

**Materials/Method:**

20 lung SBRT treatment plans using RTT were replanned with the ITV method using respiratory motion information from a 4D‐CT image acquired at the original simulation. Risk of symptomatic radiation pneumonitis was calculated for both plans using a previously derived NTCP model. Features available before treatment planning that identified significant increase in NTCP with ITV versus RTT plans were identified.

**Results:**

Prescription dose to the planning target volume (PTV) ranged from 22 to 60 Gy in 1–5 fractions. The median tumor diameter was 3.5 cm (range 2.1–5.5 cm) with a median volume of 14.5 mL (range 3.6–59.9 mL). The median increase in PTV volume from RTT to ITV plans was 17.1 mL (range 3.5–72.4 mL), and the median increase in PTV/lung volume ratio was 0.46% (range 0.13–1.98%). Mean lung dose and percentage dose–volumes were significantly higher in ITV plans at all levels tested. The median NTCP was 5.1% for RTT plans and 8.9% for ITV plans, with a median difference of 1.9% (range 0.4–25.5%, pairwise *P* < 0.001). Increases in NTCP between plans were best predicted by increases in PTV volume and PTV/lung volume ratio.

**Conclusions:**

The use of RTT decreased the risk of radiation pneumonitis in all plans. However, for most patients the risk reduction was minimal. Differences in plan PTV volume and PTV/lung volume ratio may identify patients who would benefit from RTT technique before completing treatment planning.

## 1 INTRODUCTION

“Stereotactic body radiation therapy” (SBRT) or “stereotactic ablative body radiotherapy” (SABR) refers to highly spatially precise radiation therapy with steep dose gradients delivered to an extracranial target, typically completed in 1–5 fractions with higher doses per fraction than conventional radiation therapy. For lung tumors, SBRT has emerged as an effective treatment technique for early stage lung primary malignancies as well as lung oligometastases with outcomes comparable to surgical resection.^1^, ^2^

Lung SBRT has the particular challenge of tumor respiratory motion. Some techniques for managing this include accounting for motion within target volumes, temporarily reducing motion by breath hold or abdominal compression, gating dose delivery by respiratory phase, or tracking tumor motion during treatment using fluoroscopy.^3^ One of the most common techniques requiring no breath control or imaging during treatment is expanding the target volume to the entire range of tumor motion across the respiratory cycle, known as the internal target volume (ITV). This is delineated using CT scans obtained at maximum inhalation and exhalation, or a set of scans acquired through the course of the respiratory cycle (4D‐CT). ITV technique has the disadvantage of including more normal lung tissue in the target volume, exposing it to high radiation dose. It also cannot account for unpredicted variations in tumor motion and respiratory pattern during treatment. Compensation may be achieved with larger planning target volume (PTV) margins, but this further increases the dose to normal lung tissue.^4^ To overcome these issues, real‐time tracking (RTT) techniques were developed such as the CyberKnife robotic SBRT system with Synchrony motion management (Accuray Inc., Sunnyvale CA, USA).^5^ With this system, tumor motion is tracked during treatment while the patient breathes freely without coaching. A correlation model is built between orthogonal x‐ray images acquired every 60–120 s and the positions of light emitting diodes on the patient's chest obtained by infrared camera at 26 Hz. The model is continuously updated and used to move a linear accelerator mounted on a robotic arm, anticipating target location with high accuracy.^6^ The original Synchrony system required invasive placement of gold fiducial markers near the tumor for accurate targeting, associated with risks of pneumothorax, bleeding, and additional treatment cost.^7^ The subsequently developed XSight Lung Tracking system (Accuray Inc.) allows tracking based on imaging the tumor itself, obviating the need for fiducial marker placement.^8^, ^9^ However, this technique is limited to larger and denser tumors that have adequate x‐ray contrast with normal tissue.^10^

These motion management systems were primarily designed to optimize target coverage. Less is known about their effects on normal tissue doses, and complications have not been directly compared. The most common adverse effects of lung SBRT are due to radiation of the lung parenchyma, which incites a complex reaction leading to depletion of alveolar pneumocytes, interstitial infiltration of immune cells, and fibroblast proliferation.^11^ This manifests pathologically as a continuum from subacute radiation pneumonitis to late pulmonary fibrosis, which may be clinically symptomatic.^12^ The reported incidence for symptomatic radiation pneumonitis requiring treatment after SBRT ranges from 5% to 30%.^13^, ^14^, ^15^, ^16^, ^17^, ^18^, ^19^, ^20^, ^21^, ^22^ This typically presents as a syndrome of dyspnea, cough, and low‐grade fever within 12 weeks of the completion of radiation therapy. Symptoms usually resolve with corticosteroids, although permanent pulmonary dysfunction can occur.^12^ The lungs function as a classic radiobiological “parallel organ”^23^ and symptomatic radiation pneumonitis is generally correlated with critical dose–volumes rather than maximum dose to lung tissue.^14^, ^18^, ^19^, ^22^ Normal tissue complication probability (NTCP) models based on mean lung dose have been derived to predict risk of symptomatic radiation pneumonitis from lung SBRT.^16^, ^17^, ^24^

At our institution, it is standard for all lung SBRT patients to undergo respiratory 4D‐CT imaging at simulation regardless of the intended motion management technique. This allows assessment of target and organ motion and provides a backup method if fiducial‐ or tumor‐based tracking fails. In the current study, patients originally treated using RTT were replanned using ITV volume expansion technique. Comparisons of planning parameters, lung dose–volumes, and radiation pneumonitis NTCP from a previously derived model were conducted. Classifications based on predosimetric characteristics were performed to generate practical guidelines predicting which patients could substantially benefit from real‐time tracking, without needing to perform time‐consuming replanning and NTCP calculation.

## 2 MATERIALS AND METHODS

A single institution database was used to retrospectively identify 20 SBRT treatment plans for primary or oligometastatic lung tumors. All treatments were performed on the CyberKnife system with XSight Lung Tracking RTT technique. The original treatment plans were designed to deliver the prescription dose to a planning target volume (PTV_RTT_), which was defined by an isotropic expansion from a gross tumor volume (GTV) contoured on the simulation CT. The PTV_RTT_ margin expansion was determined by the treating physician at the time of planning. Originally delivered treatment plans were used for comparisons without modification.

For our study, each patient's 8‐phase respiratory 4D‐CT images obtained at the original simulation were restored. To delineate tumor motion during a respiratory cycle, an internal target volume (ITV) was contoured by superposition of GTVs contoured on each phase, following the movement of the original GTV as much as possible. This was performed by the original treating physician at the time of initial planning if available (2 plans), or by a single physician (C.C.) retrospectively if not available. A new planning target volume (PTV_ITV_) was created using 5 × 5×8 mm (LR × AP × SI) expansion from the ITV, a previously determined appropriate planning margin for this technique.^4^

**Figure 1:**
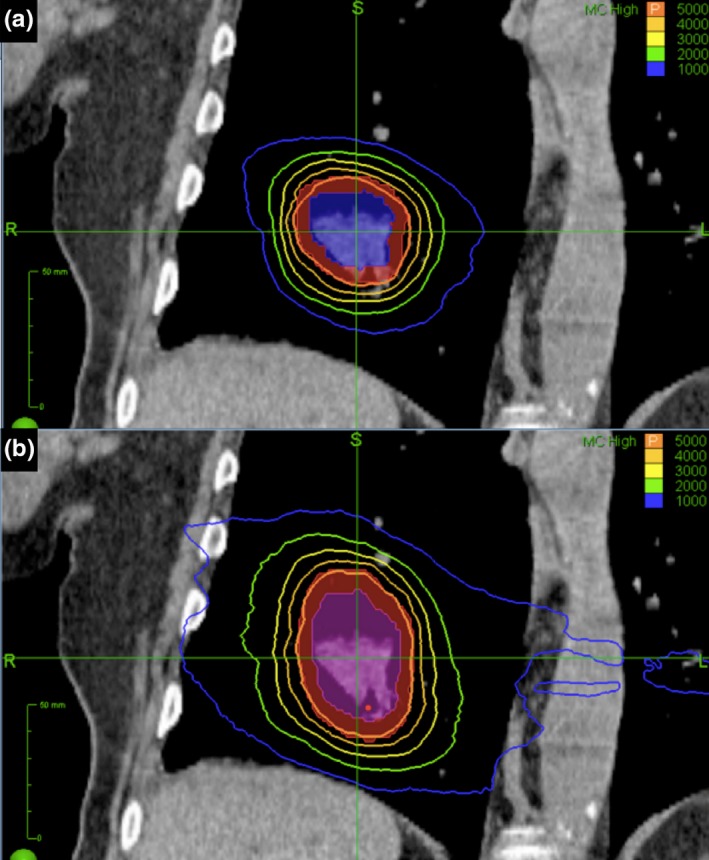
Example plan comparison with coronal slices of treatment planning CT for RTT plan (a) and ITV plan (b). GTV is shaded blue, ITV is shaded purple, and PTVs are shaded red. From RTT to ITV based‐plan, PTV volume increased 36.4 mL, PTV/lung volume ratio increased 0.69%, and NTCP increased 4.1%.

New treatment plans targeting the PTV_ITV_ were generated using the original prescription doses. Plans were designed using Accuray Multiplan software v.5.2.0. Tissue heterogeneity correction was performed by Monte Carlo algorithm with 1% uncertainty. Planning goals were for prescription dose to cover ≥95% of PTV_ITV_, and normal tissue doses to be as low as achievable while maintaining target coverage. Doses to organs at risk (spinal cord, heart, esophagus, rib/chest wall) had to meet TG‐101 constraints^25^ except for rib/chest wall, which was kept as low as possible while maintaining target coverage. Prescription isodose line ≥60% of maximum dose was used if able to maintain target coverage and normal tissue dose constraints. The PTV volumes, prescription target coverage, prescription isodose line, conformity index,^26^ total MU, estimated delivery time per fraction, and doses to organs at risk were recorded. Figure 1 depicts example RTT and ITV‐based plans from a single patient.

The volume of bilateral lungs excluding GTV was defined for each plan. Dose–volume histograms for the bilateral lung volumes were extracted with bin size 0.1 Gy. Doses were converted to the linear‐quadratic equivalent dose in 2 Gy fractions (EQD2) using α/β = 3.^27^ After conversion, the mean dose to bilateral lungs was recorded, as well as the following dose volume percentages based on previous publications identifying correlations to radiation pneumonitis risk: V2.5 Gy, V5 Gy, V10 Gy, V13 Gy, V20 Gy, V30 Gy, V40 Gy, and V50 Gy.^16^, ^17^, ^24^, ^28^ The normal tissue complication probability (NTCP) for symptomatic radiation pneumonitis requiring treatment (grade ≥2 by NCI‐CTCAE v.4) was calculated from a previously published model using the Lyman‐Kutcher‐Burman formula based on bilateral lung mean dose using TD50 = 20.8 Gy, *m* = 0.45.^17^ For patients with multiple tumors, each tumor was planned separately and doses were considered independently.

Statistical analysis was performed using non‐parametric tests in R v.3.3.2 (R Foundation for Statistical Computing).^29^ To compare treatment plan dosimetry, pairwise Wilcoxon rank‐sum tests were used. To correlate differences in NTCP to tumor and treatment features, Spearman's rank correlation coefficient was used. All statistical tests were two‐sided with a significance threshold of *P* ≤ 0.05. To create a practical guideline to identify patients before dosimetric calculation who would have a meaningfully increased risk of radiation pneumonitis with ITV versus RTT planning, an increase in NTCP of >5% was designated as “clinically significant”. This threshold was chosen by agreement that this was the highest increase in NTCP that would be accepted for plans to be considered clinically equivalent. Receiver operating characteristic (ROC) analysis was used to compare the ability of features available before dosimetric calculation (“pre‐dosimetric variables”) to predict which patients would have these increases in NTCP.

## 3 RESULTS

### 3.A Plan characteristics and NTCP

We identified 20 RTT plans delivered to 18 patients (two patients had two tumors treated by separate plans). Twelve patients were male and the median age was 73 yr (range 26–95 yr). Fourteen treatments were for lung primary tumors and six for metastatic tumors. Table 1 details the tumor locations, size, and extent of motion, as well as prescription doses (delivered and EQD2) and PTV_RTT_ margins. Two tumors were considered central by RTOG 0813 criteria (within 2 cm of the proximal bronchial tree).^30^

**Table 1:** Lung tumor SBRT plan characteristics

Plan characteristics (*n* = 20)	Number (percentage) or median (range)
Lobe	
RUL	9 (45%)
LLL	5 (25%)
LUL	2 (10%)
RML	2 (10%)
RLL	1 (5%)
Lingula	1 (5%)
Location	
Peripheral	18 (90%)
Central	2 (10%)
Total bilateral lung volume	3421 mL (2298 to 6228 mL)
GTV greatest axial diameter	3.5 cm (2.1 to 5.5 cm)
GTV volume	14.5 mL (3.6 to 59.9 mL)
ITV volume	23.3 mL (4.6 to 77.7 mL)
ITV subtract GTV volume	5.5 mL (1.0 to 22.3 mL)
Tumor motion	
Superior–inferior	4.5 mm (0 to 20 mm)
Anterior–posterior	3 mm (0 to 9 mm)
Left–right	2 mm (0 to 8 mm)
Prescription dose and fractions	
60 Gy in 5 fractions	2 (10%)
54 Gy in 3 fractions	3 (15%)
50 Gy in 5 fractions	7 (35%)
48 Gy in 4 fractions	2 (10%)
42.5 Gy in 5 fractions	1 (5%)
37.5 Gy in 3 fractions	1 (5%)
25 Gy in 1 fraction	2 (10%)
24 Gy in 1 fraction	1 (5%)
22 Gy in 1 fraction	1 (5%)
Prescription EQD2 (α/β = 3)	130 Gy (97.75 to 226.8 Gy)
Isotropic PTV_RTT_ margin	
5.0 mm	14 (70%)
2.5 mm	4 (20%)
2.0 mm	2 (10%)

Comparisons of RTT and ITV plans are in Table 2. As expected, the PTV volume was significantly larger in ITV than RTT plans (median difference 17.1 mL, range 3.5–72.4 mL, *P* < 0.001) and the ratio of the PTV volume to total bilateral lung volume (PTV/lung) was also larger (median difference 0.46%, range 0.13–1.98%, *P* < 0.001). There were no significant differences in PTV prescription coverage, prescription isodose percentages, or conformity indices. There were also no significant differences in maximum doses to the organs at risk other than the lungs (Table S1).

**Table 2:** Planning and dosimetric characteristics of ITV and RTT plans

Target	ITV		RTT		Pairwise difference (ITV − RTT)		
	Median	Range	Median	Range	Median	Range	*P*
PTV volume (mL)	60.0	21.3–160.6	33.1	6.6–88.2	17.1	3.5–72.4	<0.001
PTV/lung (%)	1.53	0.58–4.40	1.00	0.16–2.42	0.46	0.13–1.98	<0.001
PTV coverage (%)	95.5	95.0–97.0	95.6	95.0–96.7	0	−1.4 to 1.1	0.76
Rx IDL (%)	62.0	60.0–73.0	63.0	51.0–74.0	0.5	−13.0 to 10.0	0.92
Conformity index	1.08	1.01–1.19	1.10	0.98–1.36	−0.02	−0.32 to 0.10	0.19
Total MU	48,930	26,024–77,100	36,132	14,018–50,722	15,690	−8236 to 36,977	<0.001
Minutes per fraction	45.5	36–73	33	24–57	12.5	2–40	<0.001
Bilateral lung							
Mean (Gy)	8.20	3.84–18.7	5.49	1.72–11.95	1.95	0.22–7.37	<0.001
V2.5 Gy (%)	34.2	14.6–57.5	27.1	10.1–40.7	8.9	0.2–19.7	<0.001
V5 Gy (%)	22.7	8.3–41.5	18.3	5.5–32.9	3.4	−1.0 to 12.6	<0.001
V10 Gy (%)	13.6	5.3–29.0	11.0	3.5–21.3	2.0	−2.6 to 7.7	0.001
V13 Gy (%)	10.9	4.4–24.4	8.9	2.7–18.4	1.6	−2.6 to 6.3	0.004
V20 Gy (%)	7.7	3.3–16.1	6.2	1.7–13.0	1.0	−1.5 to 4.8	0.003
V30 Gy (%)	5.4	2.4–11.8	4.4	1.1–9.7	0.9	−1.0 to 3.4	0.002
V40 Gy (%)	4.2	1.9–9.5	3.4	0.8–7.8	0.8	−0.7 to 3.0	0.001
V50 Gy (%)	3.8	1.6–8.0	2.8	0.6–6.5	0.8	−0.6 to 2.6	0.001
NTCP (%)	8.9	3.5–41.1	5.1	2.1–17.2	1.9	0.4–25.5	<0.001

**Figure 2:**
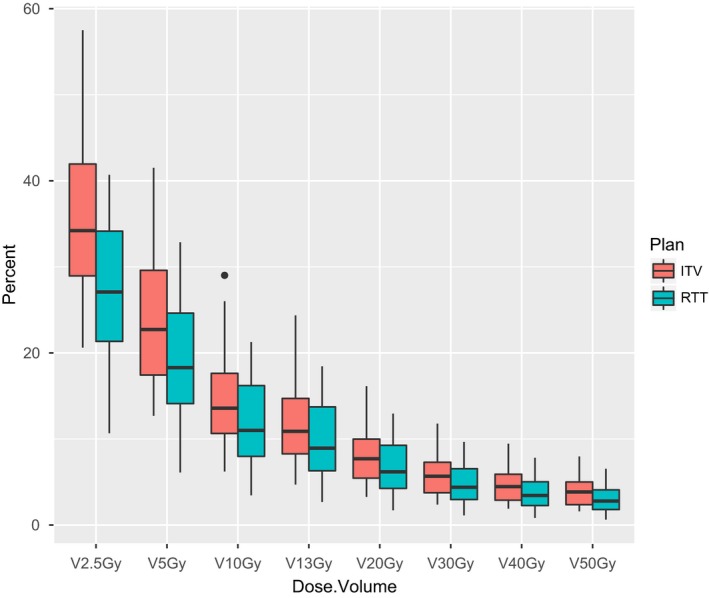
Boxplots of lung dose–volume percentages by plan type.

The mean lung dose was significantly higher for ITV plans, with a median increase in 1.95 Gy (range 0.22–7.37 Gy, *P* < 0.001). Lung dose–volume percentages were also significantly higher at every level tested, with greater increases at the lower dose–volumes (Table 2, Fig. 2). The median NTCP for RTT plans was 5.1% (range 2.1–17.2%), while the median NTCP for ITV plans was 8.9% (range 3.5–41.1%). The median RTT‐to‐ITV increase in NTCP was 1.9% (range 0.4–25.5%, *P* < 0.001). The increase in NTCP between plans was significantly correlated to the NTCP value for the ITV plan (*ρ* = 0.63, *P* = 0.004), but not to the NTCP value for the RTT plan (*ρ* = 0.32, *P* = 0.18).

### 3.B Pre‐dosimetric variable correlations to increase in NTCP

Correlations between predosimetric variables and the increase in NTCP between RTT and ITV plans are in Table 3. Increase in NTCP was most strongly correlated with the increase in PTV/lung volume ratio from RTT to ITV plan (*ρ* = 0.79, *P* < 0.001), and the increase in PTV volume from RTT to ITV plan (*ρ* = 0.74, *P* < 0.001). High correlations were also seen for PTV_ITV_/lung volume ratio (*ρ* = 0.66, *P* = 0.002), and GTV greatest axial diameter (*ρ* = 0.63, *P* = 0.003). Statistically significant but weaker correlations were also seen for other measures related to tumor size and motion such as GTV volume, ITV volume, ITV − GTV volume difference, and PTV volumes. Prescription dose, lung volume alone, and measures of tumor linear motion alone were not statistically significantly correlated with increase in NTCP between RTT and ITV plans.

**Table 3:** Spearman's rank correlation coefficient for predosimetric variables and increase in NTCP from RTT to ITV plan

Predosimetric variable	*ρ*	*P*
Prescription dose EQD2	0.25	0.29
Total lung volume	−0.23	0.32
GTV greatest axial diameter	0.63	0.003
GTV volume	0.56	0.01
ITV volume	0.56	0.01
ITV − GTV volume difference	0.52	0.02
PTV_RTT_ volume	0.46	0.04
PTV_ITV_ volume	0.59	0.008
Increase in PTV volume	0.74	<0.001
PTV_RTT_/lung volume ratio	0.43	0.06
PTV_ITV_/lung volume ratio	0.66	0.002
Increase in PTV/lung volume ratio	0.79	<0.001
Superior–inferior motion	0.20	0.39
Anterior–posterior motion	0.02	0.92
Left–right motion	−0.10	0.69

### 3.C Sensitivity analysis

The size of the PTV_RTT_ margin used in the original plans was correlated with tumor size and was potentially a confounder. A sensitivity analysis was performed limiting the comparisons to the 14 plans with an original 5 mm PTV_RTT_ margin, which tended to be used for smaller tumors. The median GTV was 12.9 mL for tumors with 5 mm PTV_RTT_ margin versus for 14.5 mL for all tumors (Tables S2–S4). The increase in NTCP from RTT to ITV plans remained significant, with a median increase in 1.2% (*P* < 0.001). The increase in PTV volume and PTV/lung volume ratio from RTT to ITV plans also remained significantly correlated with increase in NTCP (increase in PTV volume: *ρ* = 0.57, *P* = 0.03; increase in PTV/lung volume ratio: *ρ* = 0.55, *P* = 0.04).

### 3.D ROC analysis

**Figure 3:**
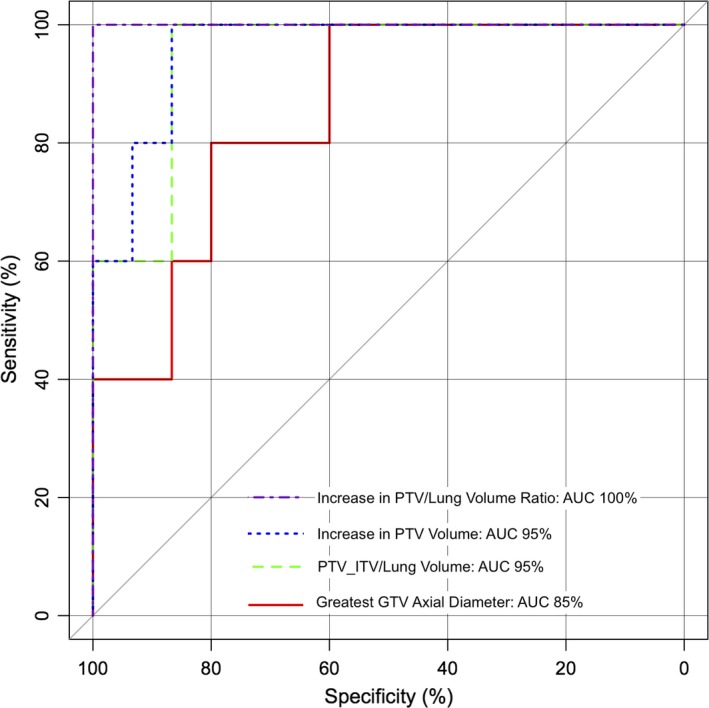
ROC curves for analyzed predosimetric variables for predicting increase in NTCP > 5%.

For the purposes of identifying tumors that would derive a clinically meaningful reduction in radiation pneumonitis risk with an RTT plan versus an ITV‐based plan, a >5% difference in NTCP was designated as “clinically significant”. Of the 20 plans, five had clinically significant increases in NTCP using ITV versus RTT. ROC analysis was used to identify predictive thresholds in the four predosimetric variables that were most highly correlated with change in NTCP from RTT to ITV plans (difference in PTV/lung volume ratio, difference in PTV volume, PTV_ITV_/lung volume ratio, and GTV greatest axial diameter). ROC curves are depicted in Fig. 3.

**Figure 4:**
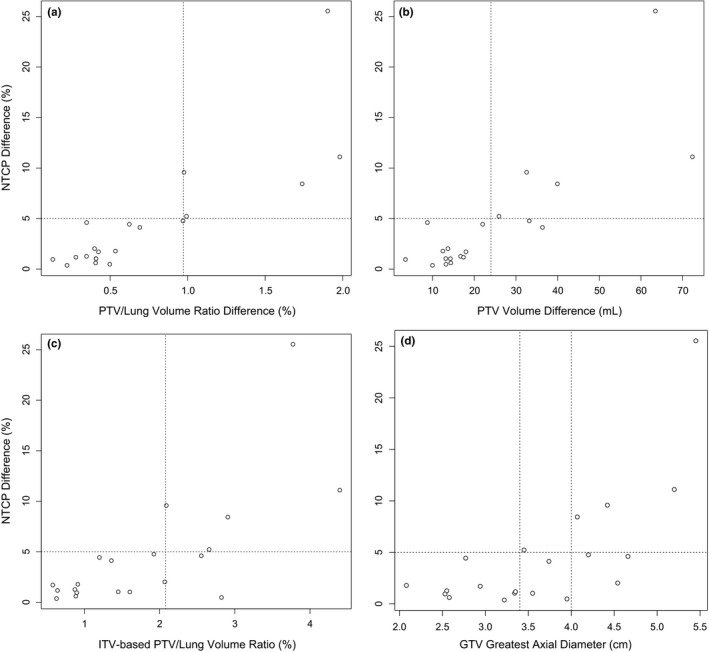
Scatterplots of increase in NTCP by increase in PTV/lung volume ratio (a), increase in PTV volume (b), PTV_ITV_/lung volume ratio (c), and GTV greatest axial diameter (d). Plots are marked at 5% increase in NTCP and at proposed classification thresholds (a: 0.973%; b: 24.0 mL; c: 2.08%; d: 3.4 cm or 4.0 cm).

Increase in PTV/lung volume ratio from RTT to ITV plan was the most accurate predictor of clinically significant increase in NTCP, with ROC area under curve (AUC) of 100%. The most sensitive threshold was an increase in PTV/lung volume ratio of 0.973% [sensitivity 100%, specificity 100%; Fig. 4(a)]. The next best predictor was the increase in PTV volume from RTT to ITV plan, with AUC 95%. The most sensitive threshold was an increase in PTV volume 24.0 mL [sensitivity 100%, specificity 87% (2 false positives); Fig. 4(b)]. The PTV_ITV_/lung volume ratio also had AUC 95%, with the most sensitive threshold being 2.08% [sensitivity 100%, specificity 87% (2 false positives); Fig. 4(c)]. The least accurate predictor of these four variables was GTV greatest axial diameter, with AUC 85%. The most sensitive threshold was diameter 3.4 cm [sensitivity 100%, specificity 60% (6 false positives); Fig. 4(d)]. Using a threshold of 4.0 cm improved the specificity [sensitivity 80% (1 false negative), specificity 80% (3 false positives); Fig. 4(d)]. However, AUC estimates were not statistically significantly different from each other.

## 4 DISCUSSION

In this series of 20 lung SBRT plans using a real‐time tracking (RTT) technique for respiratory motion management, we found that changing to an internal target volume (ITV) technique would have increased the radiation pneumonitis risk for every plan, up to 25%. However, for half of the patients this increase in normal tissue complication probability (NTCP) would have been minimal (<2%), and only 25% of patients would have experienced a “clinically significant” increase in >5%. The most predictive factors of a NTCP increase were differences between plans in PTV volume and PTV/lung volume ratio, however the PTV_ITV_/lung volume and the GTV greatest axial diameter were also strong predictors. While different PTV_RTT_ expansion margins were used for some patients, a sensitivity analysis showed that differences in PTV volume and PTV/lung volume ratio remained significant predictors of NTCP in a uniform margin subgroup. We identified thresholds in these variables to predict which patients would have no clinically significant increase in NCTP with an ITV versus an RTT based plan. These patients could be identified even before completing treatment planning, saving time needed to create multiple plans for comparison. There were otherwise no significant differences in RTT and ITV plan quality as assessed by target prescription dose coverage, conformity index, prescription isodose level, or doses to other organs at risk.

Reviewing which predosimetric variables predicted greater NTCP increase in this study (Table 3), it makes some intuitive sense that features which incorporated both target volume and motion, as well as their relation to normal lung volume, would have the greatest predictive value. Measurements of tumor volume alone (GTV volume) or motion alone (ITV − GTV volume difference, superior‐inferior motion) were less predictive, although GTV greatest axial diameter was strongly correlated. This may be due to the fact that in this dataset, smaller tumors were more often planned with a small PTV_RTT_ margin. None of the features except those based on differences between plans (increase in PTV volume, increase in PTV/lung ratio) remained significant in the uniform margin sensitivity analysis. Prescription dose was not a significant predictor within the range of doses delivered in this study, possibly because most dose/fractionation schemes are designed to have comparable toxicities. Metrics related to the PTV_ITV_ had greater correlation with the change in NTCP than those related to PTV_RTT_. This is likely due to the sigmoidal nature of the NTCP curve, as the plan with the higher NTCP closer to the steepest portion of the curve (50%) would determine more of the change. This is also demonstrated by the NTCP difference being more correlated with the NTCP from the ITV plan than the RTT plan. While the median difference in NTCP was only 2% and three‐quarters of patients had <5% difference in NTCP, there was one outlier patient with an estimated increase in NTCP 25.5% (NTCP 41% with ITV‐based plan). This female patient had a left lower lobe tumor that received 54 Gy in 3 fractions using a 2.5 mm PTV_RTT_ margin. Her total lung volume was 3335 mL, near the median. She was not an outlier on tumor size or motion, although she was near the top of the range for both (GTV 42.9 mL, second highest; 18 mm SI motion, second highest). This example demonstrates again how both tumor size and motion contribute to the estimated NTCP benefit from RTT planning.

Two similar studies on the effect of respiratory motion management on radiation pneumonitis risk were identified in the literature. Depuydt et al. compared ITV planning to RTT technique on a gimbaled SBRT system.^31^ They found a statistically significant mean improvement in NTCP of 0.69% with the RTT technique. This is smaller than the current study, however the tumor volumes were also much smaller: median 4.15 mL in that study versus 14.5 mL in the current study. A different NTCP model was also used.^32^ Kim et al. compared ITV‐based plans to respiratory gated plans in 150 patients.^33^ Gated plans reduced the mean lung dose less than 0.5 Gy (EQD2) on average, and reduced the mean lung V20 Gy by <0.5%. NTCP was not calculated, but the expected risk reduction would be small. However, the mean lung doses in that study were also low regardless of planning technique, approximately 3.0–3.5 Gy versus a median of 5.5 Gy for RTT plans and 8.2 Gy for ITV plans in the current study.

These comparisons highlight some of the limitations of this work. First, the influence of tumor size on radiation pneumonitis risk makes the results dependent on the studied population. We limited our study to patients with tumors visible on orthogonal X rays using XSight Lung Tracking technique, thus having on average larger tumors that would benefit more from RTT.^10^ Second, the estimations of radiation pneumonitis risk are dependent on the NTCP model used. We selected the model used in this study from one of the largest SBRT‐specific studies with prospectively collected toxicity data, which evaluated a variety of different dose‐fractionation schemes, α/β ratios, and NTCP models.^17^ However, to our knowledge the selected model has not been validated in a second sample and may have dependencies particular to the original group. In that study, models with a linear component for high dose per fraction radiation fell within the 95% confidence interval of the standard linear‐quadratic models.^17^ However, uncertainty remains regarding the applicability of the linear‐quadratic model to highly hypofractionated radiation therapy.^34^ Studies requiring retrospective replanning are also subject to bias. While two plans had ITVs available from the time of initial planning, the remainder were contoured retrospectively and may be different than what the original treating physician would have contoured. The RTT and ITV plans appeared to be similar quality as measured by target coverage, conformity, and heterogeneity (Table 2), but it remains possible that there were some differences in planning technique that influenced the results. Thus, while we propose some predosimetric variables that in principle could predict differences in NTCP between RTT and ITV plans, these require further validation in a larger prospective dataset before clinical use.

There were some choices made in the methodology of this study that require further explanation. First, we decided not to replan the original RTT plans with a uniform PTV_RTT_ margin for all patients. For the original plans, the treating physicians made individualized decisions on PTV margin based on tumor motion, location, and other features. Although the PTV_RTT_ margins are different, they are consistent in that they were all approved by the initial treating physicians as clinically acceptable plans. For the ITV‐based plans, one may expect to have different PTV margins than RTT due to the different motion management technique alone. Rather than adjust the PTV_ITV_ margins per patient based on the original PTV_RTT_ margin (the rationale for which may no longer apply in the ITV setting), we chose a consistent PTV_ITV_ margin based on previously published measures of intrafraction tumor motion.^4^ We then performed a sensitivity analysis limited to patients with 5 mm PTV_RTT_ margins, which did not substantially alter our findings. We also made a choice to designate an NTCP difference of >5% as a “clinically significant” difference between plans. While somewhat arbitrary, identifying thresholds like this can be useful for clinical decision‐making. The authors agreed that this is the highest difference in NTCP between plans that would be acceptable to consider them clinically equivalent. In the relevant range of NTCP the ±1 SD confidence interval of the model used is approximately ±2.5%, therefore any predicted changes in NTCP below 5% may also be less reliable.^17^ The purpose of identifying predosimetric variables that predict difference in NTCP was to help clinicians to easily identify which patients would substantially benefit from RTT technique. While the most accurate approach would be to simply create both RTT and ITV‐based plans and compare dosimetry, in our experience it can take several hours to create a single lung SBRT plan. The variables proposed can be obtained with contouring only. Although not addressed in this study, patients who would not benefit from RTT technique may be also good candidates for an ITV‐based plan on a different system, thereby saving use of CyberKnife as a limited resource.

There are several factors besides pneumonitis risk that must be considered when choosing a motion management technique. If the target is close to organs at risk such as the heart or esophagus, greater motion management may be needed just to achieve these planning constraints. In this study there were no statistically significant differences in maximum doses to other organs by treatment technique across the group (Table S1). All planning constraints were met, and some ITV‐based plans had even lower maximum organ doses than the matching RTT plans, likely due to differences in planning techniques and optimization goals. The lack of significant differences across the group is also due to tumors being in various locations throughout the lungs. However, as can be seen from the table, individual patients did have differences in organ maximum doses that may approach clinical significance. Target accuracy may also be affected by motion management technique. In the CyberKnife system, alignment is performed on the spinal column when using ITV‐based planning and the target itself is not localized prior to or during treatment. Tumors further away from the spine thus have greater targeting errors, and the application of this technique should be carefully evaluated on a case‐by‐case basis. A preliminary study suggests that using spine alignment as surrogate for lung tumor localization may result in local misses and ultimately decrease tumor control.^35^ ITV‐based planning also assumes that 4D‐CT adequately captures all tumor motion, although motion can vary widely within and between fractions.^36^ Motion management technique also affects treatment delivery time. We found a median decrease in 12.5 min per fraction for RTT plans due to smaller target volume and lower MU count (Table 2). However, this does not take into account RTT pretreatment imaging and correlation model building which increase the total “on table” time, particularly for patients with irregular breathing patterns. It is reasonable to assume that the total resource utilization time may be actually higher for RTT than ITV‐based plans. Furthermore, ITV‐based plans can be delivered using volumetric arc planning, which is much faster than CyberKnife and allows 3D target localization. Treatment indications and comorbid conditions also must be considered. Lung SBRT for oligometastatic disease may be given for prophylaxis or palliation of symptoms, and treatment‐related toxicities may be considered less acceptable from a risk‐benefit perspective. Oligometastatic patients are also more likely to receive radiation to multiple lung tumors increasing total lung dose, and to receive systemic agents that may also increase pneumonitis risk. Two patients in this study had multiple tumors treated using SBRT, however, this was not taken into account when calculating radiation pneumonitis risk and may be expected to raise it substantially.

Thus, while also considering these other factors, the capability to identify patients who would benefit from RTT is valuable. If they would benefit, clinicians may recommend attempts at XSight lung tracking, fiducial implantation, or other strategies for motion management. If they would not benefit, clinicians could opt for ITV‐based treatment either on the Cyberknife or a conventional linear accelerator. In the present study we both quantify the range of expected differences in radiation pneumonitis risk between motion management techniques and propose some variables that may predict this benefit without requiring dosimetric calculation.

## 5 CONCLUSION

The use of a real‐time tumor tracking technique decreased radiation pneumonitis risk for all patients, although for most this absolute risk reduction was small (<5%). Difference between plans in the ratio of the target volume to total lung volume was the best predosimetric predictor of whether patients would derive a clinically significant benefit from real‐time tumor tracking technique. Prospective data collection in a larger sample would validate the use of this NTCP model and the estimated effect of different respiratory motion management techniques.

## CONFLICTS OF INTEREST

None.

## Supporting information


**Table S1.** Maximum doses to non‐lung OARs for ITV and RTT plans.

**Table S2.** Sensitivity analysis: characteristics of 14 plans with PTV_XLT_ margin 5.0 mm for sensitivity analysis.

**Table S3.** Sensitivity analysis: dosimetric comparison of 14 plans with PTV_RTT_ margin 5.0 mm.

**Table S4.** Sensitivity analysis: correlation of predosimetric variables to change in NTCP risk for 14 plans with PTV_RTT_ margin 5.0 mm.
