# Supplementary material for: Influence of respiratory motion management technique on radiation pneumonitis risk with robotic stereotactic body radiation therapy
Source: J Appl Clin Med Phys. 2018 Apr 26;19(4):48–57. doi: 10.1002/acm2.12338 (PMC6036380; doi:10.1002/acm2.12338)
Supplement: Supplementary file 1 — Table S1. Maximum doses to non‐lung OARs for ITV and RTT plans. [file ACM2-19-48-s001.docx]

Supplementary Table S1.

|  | **ITV** | |  | **RTT** | |  | **Pairwise Difference**  **(ITV – RTT)** | | |
| --- | --- | --- | --- | --- | --- | --- | --- | --- | --- |
| **Maximum Dose**  **to OAR** | **Median** | **Range** |  | **Median** | **Range** |  | **Median** | **Range** | ***p*** |
| Spinal Cord (Gy) | 6.03 | 2.55 – 11.98 |  | 4.93 | 1.22 – 13.63 |  | 1.06 | -458 – 501 | 0.08 |
| Heart (Gy) | 10.77 | 0.78 – 26.18 |  | 10.19 | 0.53 –32.28 |  | 1.22 | -1472 – 702 | 0.84 |
| Esophagus (Gy) | 8.33 | 3.21 – 14.70 |  | 5.42 | 0.78 – 20.92 |  | 1.16 | -622 – 833 | 0.09 |
| Chest Wall (Gy) | 42.67 | 14.34 – 73.52 |  | 40.16 | 12.14 – 67.38 |  | 2.29 | -1704 – 1383 | 0.17 |

*OAR = organ at risk. Maximum dose to 0.035 cc. Doses are per plan, not EQD2.*
