# Supplementary material for: Influence of respiratory motion management technique on radiation pneumonitis risk with robotic stereotactic body radiation therapy
Source: J Appl Clin Med Phys. 2018 Apr 26;19(4):48–57. doi: 10.1002/acm2.12338 (PMC6036380; doi:10.1002/acm2.12338)
Supplement: Supplementary file 2 — Table S2. Sensitivity analysis: characteristics of 14 plans with PTVXLT margin 5.0 mm for sensitivity analysis. [file ACM2-19-48-s002.docx]

Supplementary Table S2. Sensitivity analysis: characteristics of 14 plans with PTV_XLT_ margin 5.0 mm for sensitivity analysis

| **Plan Characteristics (*n* = 14)** | **Median (range)** |
| --- | --- |
| GTV Greatest Axial Diameter | 3.4 cm (2.5 to 4.7 cm) |
| GTV Volume | 12.9 mL (3.8 to 25.9 mL) |
| ITV Volume | 19.5 mL (7.7 to 33.6 mL) |
| ITV Subtract GTV Volume | 5.5 mL (1.3 to 18.8 mL) |
