# Supplementary material for: Influence of respiratory motion management technique on radiation pneumonitis risk with robotic stereotactic body radiation therapy
Source: J Appl Clin Med Phys. 2018 Apr 26;19(4):48–57. doi: 10.1002/acm2.12338 (PMC6036380; doi:10.1002/acm2.12338)
Supplement: Supplementary file 3 — Table S3. Sensitivity analysis: dosimetric comparison of 14 plans with PTVRTT margin 5.0 mm. [file ACM2-19-48-s003.docx]

Supplementary Table S3. Sensitivity analysis: dosimetric comparison of 14 plans with PTV_RTT_ margin 5.0 mm

|  | **ITV** | |  | **RTT** | |  | **Pairwise Difference**  **(ITV – RTT)** | | |
| --- | --- | --- | --- | --- | --- | --- | --- | --- | --- |
| **Target** | **Median** | **Range** |  | **Median** | **Range** |  | **Median** | **Range** | ***p*** |
| PTV Volume (mL) | 51.3 | 24.0 – 74.9 |  | 33.1 | 16.9 – 61.7 |  | 14.3 | 3.5 – 36.4 | <0.001 |
| PTV/Lung (%) | 1.40 | 0.62 – 2.82 |  | 0.86 | 0.36 – 2.33 |  | 0.41 | 0.13 – 0.99 | <0.001 |
| PTV Coverage (%) | 95.6 | 95.0 – 97.0 |  | 95.6 | 95.2 – 96.7 |  | 0.0 | -1.4 – 1.1 | 0.90 |
| Rx IDL (%) | 62.0 | 60.0 – 65.0 |  | 62.5 | 52.0 – 69.0 |  | 0.5 | -9.0 – 9.0 | 0.92 |
| Conformity Index | 1.08 | 1.01 – 1.19 |  | 1.07 | 0.98 – 1.30 |  | 0.01 | -0.17 – 0.10 | 0.76 |
| Total MU | 46199 | 29360 - 73971 |  | 34068 | 14018 - 50143 |  | 16890 | -8236 – 36980 | 0.002 |
| Minutes per Fraction | 44 | 36 – 73 |  | 33 | 24 - 39 |  | 12.5 | 2 - 40 | 0.001 |
| **Bilateral Lung** |  |  |  |  |  |  |  |  |  |
| Mean (cGy) | 808 | 384 – 1061 |  | 549 | 306 – 973 |  | 132 | 22 - 376 | <0.001 |
| V2.5Gy (%) | 32.7 | 21.7 – 51.5 |  | 27.1 | 16.6 – 38.6 |  | 5.9 | 0.2 – 14.1 | <0.001 |
| V5Gy (%) | 21.8 | 12.7 – 33.6 |  | 18.3 | 10.8 – 29.2 |  | 2.2 | -1.0 – 7.6 | <0.001 |
| V10Gy (%) | 12.4 | 6.2 – 19.2 |  | 11.0 | 5.4 – 19.5 |  | 1.4 | -2.6 – 6.1 | 0.05 |
| V13Gy (%) | 10.1 | 4.7 – 15.1 |  | 8.9 | 4.3 – 16.1 |  | 1.2 | -2.6 – 4.6 | 0.07 |
| V20Gy (%) | 7.3 | 3.3 – 11.6 |  | 6.1 | 3.0 – 11.1 |  | 0.9 | -1.5 – 3.6 | 0.05 |
| V30Gy (%) | 5.4 | 2.4 – 8.8 |  | 4.1 | 2.1 – 7.8 |  | 0.8 | -1.0 – 2.8 | 0.04 |
| V40Gy (%) | 4.2 | 1.9 – 7.2 |  | 3.3 | 1.7 – 6.2 |  | 0.6 | -0.7 – 2.5 | 0.04 |
| V50Gy (%) | 3.5 | 1.6 – 6.1 |  | 2.7 | 1.4 – 5.2 |  | 0.5 | -0.6 – 2.2 | 0.02 |
| NTCP (%) | 8.7 | 3.5 – 13.8 |  | 5.1 | 2.9 – 11.9 |  | 1.2 | 0.4 – 5.2 | <0.001 |
