# Supplementary material for: Influence of respiratory motion management technique on radiation pneumonitis risk with robotic stereotactic body radiation therapy
Source: J Appl Clin Med Phys. 2018 Apr 26;19(4):48–57. doi: 10.1002/acm2.12338 (PMC6036380; doi:10.1002/acm2.12338)
Supplement: Supplementary file 4 — Table S4. Sensitivity analysis: correlation of predosimetric variables to change in NTCP risk for 14 plans with PTVRTT margin 5.0 mm. [file ACM2-19-48-s004.docx]

Supplementary Table S4. Sensitivity analysis: correlation of pre-dosimetric variables to change in NTCP risk for 14 plans with PTV_RTT_ margin 5.0 mm

| **Pre-Dosimetric Variable** | ***rho*** | ***p*** |
| --- | --- | --- |
| Prescription Total Dose | -0.21 | 0.47 |
| Total Lung Volume | -0.19 | 0.52 |
| GTV Greatest Axial Diameter | 0.42 | 0.14 |
| GTV Volume | 0.38 | 0.18 |
| ITV Volume | 0.43 | 0.12 |
| ITV – GTV Volume Difference | 0.42 | 0.14 |
| PTV_RTT_ Volume | 0.31 | 0.27 |
| PTV_ITV_ Volume | 0.45 | 0.11 |
| Increase in PTV Volume | 0.57 | 0.03 |
| PTV_RTT_/Lung Volume Ratio | 0.26 | 0.37 |
| PTV_ITV_/Lung Volume Ratio | 0.42 | 0.14 |
| Increase in PTV/Lung Volume Ratio | 0.55 | 0.04 |
| Superior-Inferior Motion | -0.05 | 0.87 |
| Anterior-Posterior Motion | 0.43 | 0.12 |
| Left-Right Motion | 0.27 | 0.34 |
